# Multiple Regulatory Roles of the Mouse Transmembrane Adaptor Protein NTAL in Gene Transcription and Mast Cell Physiology

**DOI:** 10.1371/journal.pone.0105539

**Published:** 2014-08-25

**Authors:** Iva Polakovicova, Lubica Draberova, Michal Simicek, Petr Draber

**Affiliations:** Department of Signal Transduction, Institute of Molecular Genetics, Academy of Sciences of the Czech Republic, Prague, Czech Republic; University of Iowa, United States of America

## Abstract

Non-T cell activation linker (NTAL; also called LAB or LAT2) is a transmembrane adaptor protein that is expressed in a subset of hematopoietic cells, including mast cells. There are conflicting reports on the role of NTAL in the high affinity immunoglobulin E receptor (FcεRI) signaling. Studies carried out on mast cells derived from mice with NTAL knock out (KO) and wild type mice suggested that NTAL is a negative regulator of FcεRI signaling, while experiments with RNAi-mediated NTAL knockdown (KD) in human mast cells and rat basophilic leukemia cells suggested its positive regulatory role. To determine whether different methodologies of NTAL ablation (KO vs KD) have different physiological consequences, we compared under well defined conditions FcεRI-mediated signaling events in mouse bone marrow-derived mast cells (BMMCs) with NTAL KO or KD. BMMCs with both NTAL KO and KD exhibited enhanced degranulation, calcium mobilization, chemotaxis, tyrosine phosphorylation of LAT and ERK, and depolymerization of filamentous actin. These data provide clear evidence that NTAL is a negative regulator of FcεRI activation events in murine BMMCs, independently of possible compensatory developmental alterations. To gain further insight into the role of NTAL in mast cells, we examined the transcriptome profiles of resting and antigen-activated NTAL KO, NTAL KD, and corresponding control BMMCs. Through this analysis we identified several genes that were differentially regulated in nonactivated and antigen-activated NTAL-deficient cells, when compared to the corresponding control cells. Some of the genes seem to be involved in regulation of cholesterol-dependent events in antigen-mediated chemotaxis. The combined data indicate multiple regulatory roles of NTAL in gene expression and mast cell physiology.

## Introduction

Activation of mast cells upon exposure to antigen (Ag) is one of the major events in the allergic reaction. It is initiated by Ag-mediated aggregation of the high-affinity immunoglobulin (Ig) E receptor (FcεRI) armed with Ag-specific IgE, and results in degranulation leading to the release of a number of preformed allergy mediators such as histamine, serotonin, proteases, preformed cytokines, and proteoglycans. Mast cell activation also leads to the synthesis and release of numerous compounds like cytokines and those formed by arachidonic acid metabolism [1]. The first biochemically well-defined step in FcεRI signaling is tyrosine phosphorylation of the immunoreceptor tyrosine-based activation motifs (ITAMs) in the FcεRI β and γ subunits by Src family kinase LYN [2], [3]. Phosphorylation of the ITAMs leads to the recruitment and activation of SYK kinase, which phosphorylates tyrosine residues of numerous proteins involved in the intracellular signaling pathways, including two transmembrane adaptor proteins (TRAPs), linker for activation of T cells (LAT) and non-T cell activation linker (NTAL; also called linker for activation of B cells or LAT2). Both these TRAPs possess multiple sites of tyrosine phosphorylation and act as scaffolds for recruitment of various cytosolic adaptors and effector proteins [4]–[6].

NTAL is expressed in hematopoietic cells such as B cells, natural killer cells, dendritic cells, monocytes, and mast cells but not in resting T cells. NTAL is the product of human WBSCR5 gene located on chromosome 7 encoding a 243 amino acids protein. Its murine ortholog contains 203 amino acids, has a molecular weight of approximately 25 kD and is encoded by a gene located on chromosome 5 [7], [8]. NTAL contains a short extracellular domain, a transmembrane domain and a cytosolic tail which possesses a CxxC motif responsible for palmitoylation of the protein and its targeting to detergent-resistant plasma membrane microdomains. The cytoplasmic domain contains 10 tyrosines which are potential targets for tyrosine kinases. NTAL is structurally similar to another TRAP, LAT; after phosphorylation both molecules are capable of binding a number of cytoplasmic signaling molecules including GRB2, SOS1, GAB1 and C-CBL. NTAL, unlike LAT, is however unable to directly bind the phospholipase Cγ1 [7], [8].

Previously we and others showed that bone marrow-derived mast cells (BMMCs) from *Ntal^-/-^* mice were hyper-responsive to FcεRI stimulation [9], [10], whereas BMMCs from *Lat^-/-^* mice were hypo-responsive [11]. Interestingly, loss of both NTAL and LAT caused stronger inhibitory effect on FcεRI-mediated degranulation than loss of LAT alone. This suggested that NTAL could also have a positive regulatory role in FcεRI signaling, manifested only in the absence of LAT [9], [10]. In contrast to studies with cells from mice with NTAL knock out (KO), NTAL knockdown (KD) by RNAi in human mast cells [12] and also in rat basophilic leukemia cells [13] resulted in impaired degranulation; it implies that NTAL has positive regulatory roles in these cells even in the presence of LAT.

To rigorously examine the regulatory role(-s) of NTAL in murine mast cells signaling and to test the contribution of compensatory developmental alterations in mast cells from NTAL KO mice, we prepared BMMCs with NTAL KO or KD and the corresponding controls and cultured them under comparable well-defined conditions. For functional comparison of mast cells with NTAL KO or KD we examined several parameters characteristic for FcεRI signaling including degranulation, calcium mobilization, tyrosine phosphorylation of LAT and ERK, depolymerization of filamentous (F) actin, and chemotaxis. The results obtained with the NTAL KD BMMCs were very similar to those of NTAL KO cells and thus support the notion that in murine mast cells NTAL is predominantly a negative regulator of FcεRI signaling and that compensatory developmental alteration do not contribute to this phenotype.

To gain a better understanding of the genes that are regulated through NTAL-dependent pathways, we further examined the gene expression profiles of resting and Ag-activated BMMCs with NTAL KO or KD and corresponding controls. Several genes have been identified that differ by a factor of 1.8 and higher in their expression in resting and FcεRI-activated NTAL-deficient cells when compared to wild type (WT) cells. Through gene ontology analysis we identified a subset of NTAL-dependent genes, which were related to metabolism and biosynthetic processes. Further analysis showed that some of the genes could be involved in regulation of cholesterol-dependent events in chemotaxis towards antigen.

## Materials and Methods

### Cells and their activation

Bone marrow cells were isolated from femurs and tibias of 8–12 week-old WT or NTAL KO mice (males and females) of C57BL6 background [9]. Mice were bred and maintained in specific pathogen free facility of the Institute of Molecular Genetics and used in accordance with the Institute guidelines. The protocol, including killing mice by decapitation, was approved by the Institutional Animal Care and Use Committee (Permit number 12135/2010-17210). All efforts were made to minimize suffering. The cells were cultured for 6-8 weeks in mast cell medium [Iscove's modified Dulbecco's medium supplemented with 10% fetal calf serum (FCS), penicillin, streptomycin, 2-mercaptoethanol, recombinant interleukin (IL)-3 (20 ng/ml; Peprotech), and mouse stem cell factor (SCF; 40 ng/ml; Peprotech)]. In some experiments BMMCs were cultured for the indicated time intervals in mast cell medium supplemented with 10% cholesterol-depleted FCS (see below) instead of FCS. For activation, BMMCs (6×10^6^/ml) were sensitized in medium without SCF and IL-3, but supplemented with trinitrophenyl (TNP)-specific IgE (IGEL b4 1 monoclonal antibody; 1 µg/ml). After 4 hours the cells were washed in buffered salt solution (BSS; 20 mM HEPES, pH 7.4, 135 mM NaCl, 5 mM KCl, 1.8 mM CaCl_2_, 5.6 mM glucose, and 1 mM MgCl_2_) supplemented with 0.1% bovine serum albumin (BSA) and stimulated with various concentrations of Ag (TNP-BSA conjugate) and/or SCF. Degree of degranulation was determined by measuring the release of β-glucuronidase from the activated cells as previously described [14].

### Cholesterol-depleted FCS and cholesterol determination

FCS was cleared of cholesterol and other lipids by organic extraction as described [15]. Briefly, 100 ml of FCS was mixed with 200 ml of a mixture n-butanol and diisopropylether at a 40∶60 (v/v) ratio. After incubation at room temperature (22°C) for 1 hour in dark, the mixture was centrifuged at 22°C for 15 minutes at 6000 rpm in a JA-10 rotor, Beckman Coulter. The bottom phase containing delipidated serum was recovered and lyophilized. The resulting dry pellet was resuspended in 100 ml deionized H_2_O and filter-sterilized through 0.22 µm filter. Concentration of cholesterol in serum and cell samples was determined by the Amplex Red Cholesterol Assay kit (Life Technologies) according to the manufacturer's instruction. Using this kit, no remaining cholesterol was detectable in delipidated serum. This indicates that cholesterol concentration was reduced from ∼80 µg/ml to <15 ng/ml. For determination of cellular cholesterol, frozen cell pellet containing 0.35×10^6^ cells was lysed in 30 µl of ice cold lysis buffer (10 mM EDTA, 100 mM NaCl, 10 mM Tris-HCl, pH 7.5, 0.2% SDS, 0.5% Nonidet P-40, 0.5% sodium deoxycholate) and 2 µl aliquots were analyzed for cholesterol content using the same kit as above. Protein content in the lysates was determined by BCA protein assay kit (Pierce Chemical Co.) and the amounts of cholesterol were normalized to protein contents.

### Antibodies and immunoblotting

All antibodies were purchased from Santa Cruz, except for anti-NTAL (NAP-07; Exbio), anti-phospho-LAT (Upstate Biotechnology) and anti-LAT [16]. Cells were solubilized for 30 minutes in ice-cold lysis buffer containing 50 mM Tris-HCl, pH 7.4, 150 mM NaCl, 2 mM EDTA, 10 mM β-glycerophosphate, 1 mM Na_3_VO_4_, 1 mM PMSF, 1 µg/ml aprotinin, 1 µg/ml leupeptin, 0.2% Triton X-100, 1% Nonidet P-40 and 1% n-dodecyl-β-D-maltoside. After centrifugation (15 minutes at 7.000×g at 4°C), proteins in postnuclear supernatants were size fractionated by SDS-polyacrylamide gel electrophoresis and analyzed by direct immunoblotting. Immunoblots were quantified by Luminiscent Image Analyzer LAS 3000 (Fuji Photo Film Co.) and further analyzed by AIDA image analyzer software (Raytest).

### Measurement of free cytoplasmic Ca^2+^

Concentration of free cytoplasmic Ca^2+^ [Ca^2+^]_i_ was determined using cells labeled with Fura-2-AM (Molecular Probes) as described [9]. Ca^2+^ levels were monitored by means of fluorescence reader Infinite M200 (Tecan) with excitation wavelengths of 340 and 380 nm, and emission wavelength of 510 nm.

### Lentiviral vectors and gene transduction

A set of 5 shRNA constructs designed to target murine NTAL (GenBank accession number NM_020044) and cloned into the pLKO.1 vector was purchased from Open Biosystems to prepare BMMCs with NTAL KD. From these five shRNA constructs (TRCN0000127239, NTAL KD 1; TRCN0000127240, NTAL KD 2; TRCN0000127241, NTAL KD 3; TRCN0000127242, NTAL KD 4; TRCN0000127243, NTAL KD 5), the NTAL KD 3 and NTAL KD 5 showed reproducibly the highest reduction of NTAL protein expression in target cells and were used in most of the experiments with similar results. In all experiments in this study we obtained similar data with these two constructs and therefore the data were pooled and are referred to as NTAL KD. For microarray gene expression analysis and related qPCR validation, cells with the NTAL KD 5 construct were used.

Lentiviral transduction was performed as described previously [17]. Briefly, 21 µl ViraPower packaging mix (Invitrogen Life Technologies) and 14 µg NTAL shRNA or pLKO.1 empty vector as a negative control [in 1.4 ml medium Opti-MEM (Invitrogen)] were co-transfected into 293T17 packaging cells in the presence of 84 µl Lipofectamine 2000 (Invitrogen) or 105 µl polyethylenimine (25 kD, linear form, 1 µg/ml; Polysciences). After 2–3 days, the culture supernatants were centrifuged to pellet the viruses, which were then used to infect NTAL WT BMMCs. Stable transfectants were selected in puromycin (5 µg/ml; InvivoGen). After one week of selection, cells were analyzed for NTAL expression by immunoblotting. Before the tests, cells were transferred for 2–3 days into fresh media without puromycin.

### F-actin assay

Total amount of F-actin in nonactivated and activated cells was determined by flow cytometry. Cells in 96-well plates (50 000 cells per well) were exposed to various stimuli at 37°C, fixed with 3% paraformaldehyde in phosphate buffered saline and then permeabilized and stained in a single step by a mixture of lysophosphatidylcholine (200 µg/ml) and 1000x diluted Alexa Fluor 488-phalloidin (Molecular Probes) in phosphate buffered saline. Fluorescence intensity was measured with the help of LSRII flow cytometer (Becton Dickinson). Acquired data were analyzed using FlowJo software (Tree Star Inc).

### Cell Spreading

Wells (6 mm in diameter) of 8-well multitest slides (MP Biomedicals) were coated with fibronectin (Sigma Aldrich) and Cell-Tak (BD Biosciences) as described [18]. IgE-sensitized BMMCs were seeded on fibronectin/Cell-Tak-coated wells and allowed to attach for 1 hour. The attached cells were activated for 30 minutes at 37°C with Ag and/or SCF in BSS-0.1% BSA, fixed with 3% paraformaldehyde in glutamate buffer-EGTA (GBE; 137 mM K-glutamate, 2 mM MgCl_2_, 3 mM EGTA, and 20 mM PIPES-NaOH, pH 6.8) supplemented with 4% polyethylene glycol, molecular weight 3200, and then permeabilized and stained by one-step exposure to lysophosphatidylcholine (200 µg/ml) and 100x diluted Alexa Fluor 488-phalloidin in GBE. Coverslips were mounted in glycerol-based mounting medium containing Mowiol 4-88 reagent (Calbiochem AG) and Hoechst 33258 (3 µg/ml; Sigma). Fluorescent images (25 images/well) were automatically collected using Olympus IX70 inverted microscope (objective LUCPLFLN Ph1 20x) equipped with motorized stage and ScanR acquisition software (Olympus). Cell area was analyzed using ScanR analysis software (Olympus). At least 500 cells were evaluated in each test.

### RNA preparation

Total RNA was isolated from 3×10^6^ resting or Ag-activated (100 ng/ml TNP-BSA in BSS-0.1% BSA, 37°C, 2 hours) BMMCs using the RNeasy mini kit (Qiagen) according to the manufacturer's protocol. Three biological replicates were carried out with each cell type: NTAL KO, WT, NTAL KD, and mock (empty pLKO.1 vector) infected WT cells (referred to as WT pLKO). Cells in each group were cultured in parallel for 24 hours in complete media deprived of SCF and then sensitized with TNP-specific IgE in IL-3- and SCF-deprived medium for 4 hours. After removal of unbound IgE by washing, cell suspensions were divided into 2 aliquots; one activated for 2 hours with Ag (100 ng/ml) and the other incubated without Ag (nonactivated control cells; 0 hours). RNA was isolated from all 24 samples and processed under identical conditions. For RNA quantification, the absorption at 260 nm was measured using NanoDrop spectrophotometer N-1000 (NanoDrop Technologies).

### Microarray gene-expression profiling and data analysis

Preparation of cRNA, hybridization and gene expression profiling was done by an Affymetrix authorized service provider (AROS Applied Biotechnology A/S) using the Affymetrix GeneTitan HT MG-430 PM 24-array plate with the 3′ IVT express labeling kit according to the manufacturer's protocol. Briefly, following fragmentation, 6.5 µg aliquots of cRNA were hybridized for 16 hours at 45°C on the Affymetrix array plate using the Affymetrix GeneTitan system. The array plate was washed, stained and scanned using the Affymetrix GeneTitan system with GCOS 1.4 software. One of the 24 samples analyzed, activated NTAL KO replicate 2, failed during the hybridization, wash and scan step and was removed. Data analysis was carried out by importing raw data CEL files into Genomic Suite Software Partek 6.4 (version 6.09.0602), where the Robust Multichip Analysis was used for background correction. Using the same software, principal component analysis (PCA) of the normalized microarray expression values was performed as a visualization technique to determine the similarity in the data. Lists of significantly upregulated or downregulated gene transcripts were created based on a change greater than 1.8-fold and false discovery rate (FDR) <0.1, with one exception in which activated cells were compared with nonactivated cells (fold change >4; FDR <0.05). Only well annotated probe sets (Affymetrix annotation version from July 2011) are listed in the tables. The microarray study was performed according to the standards of the Microarray Gene Expression Society. Data complying with the Minimum Information About Microarray Experiments (MIAME; [19]) were uploaded in the NCBIs Gene Expression Omnibus (GEO) database and are available under the accession number GSE40731.

### Reverse transcription quantitative PCR (RT-qPCR)

cDNA was synthesized using mouse Moloney leukemia virus reverse transcriptase (Invitrogen) according to manufacturer's instructions. For reverse transcription, 0.3 µg aliquots of total RNA were used from the same samples, which were used for microarray analysis. qPCRs were performed using a PCR mastermix supplemented with 0.2 M trehalose, 1 M 1,2-propanediol and SYBR green I as described [20]. Ten μl reaction volumes in 384-well plates sealed with LightCycler 480 sealing foil (Roche Diagnostics) were processed in LightCycler 480 (Roche Diagnostics) under the following cycling conditions: initial 3 minutes denaturation at 95°C, followed by 50 cycles at 95°C for 10 s, 60°C for 20 s and 72°C for 20 s. Melting curve analysis was carried out from 72°C to 97°C with 0.2°C increments; Ct values for each sample were determined by automated threshold analysis. Primer pairs used for cDNA amplification are listed in Table 1. Data were normalized to a housekeeping GAPDH mRNA. The qPCRs for each of the biological triplicates was performed in quadruplicates.

**Table 1:** Primers used in quantitative RT-PCR.

Gene	Primer	Primer sequence (5′-3′)	Product size (bp)
Dusp5	Forward Reverse	GGGGTATGAGACCTTCTACTCAC GGAGGCTTCTCTCGCCTTC	92
Fdps	Forward Reverse	GCCATCAACGACGCTCTGCT ATGGCCCTGGGGTGCTGTCA	165
Idi1	Forward Reverse	AGCTTCTAGCGGAGATGTGTA CAGCAACTATTGGTGAAACAACC	214
Klhl24	Forward Reverse	TGAAATGGACCCCAAATCTCTG GGACAGCTCGATGGCATGG	170
Lss	Forward Reverse	TCGTGGGGGACCCTATAAAAC CGTCCTCCGCTTGATAATAAGTC	104
Mki67	Forward Reverse	AGAGCCTTAGCAATAGCAACG GTCTCCCGCGATTCCTCTG	145
Mlec	Forward Reverse	GAGGAGGGCGACTATGTGC CATTCAACCGGACATCAAACAC	88
N4bp2l1	Forward Reverse	CCAGAGAAGAGCAAGGAAAGC TCGGGTTCCCGGAATATAACTT	141
Nt5dc2	Forward Reverse	GCCTCATGTACCAGTGGATCG GACCTACCATGTGCCGCATAC	165
Otub2	Forward Reverse	TTCTACAGGGCCTTAGGCTATT AGCACACGCTCTTTGAACTTG	83
Plau	Forward Reverse	ATGGAAATGGTGACTCTTACCGA TGGGCATTGTAGGGTTTCTGA	106
Pmvk	Forward Reverse	TGAGGCTTGAGGGGACGAGCA TTGCTGACCTCGGCTGGGACT	90
Sdf4	Forward Reverse	TGTGGTCTAGCTGCTCATGG TGTGGTCTAGCTGCTCATGG	103
Slain1	Forward Reverse	TCACCAGAGAAATCCCCGAGT CCCCTGGAGGTATAACCTTGC	125
Spink4	Forward Reverse	GCCTTGTTTTCCCCAGAATGCCCTT CGGGTCAGGCAAAGGTGGCA	130
Tmcc3	Forward Reverse	CCCTCAGCCTACCCCTGAA CGCATTGAGTTTGACCTTGTGG	104
NTAL WT	Forward	TGGACACAGCTCCAAACAAG	197
NTAL KO	Forward	CTGCTCACCAGAGTCAGTGG	160
NTAL WT/KO	Reverse	GGAATCGTCGTCTTCTGAGG	
GAPDH	Forward Reverse	AACTTTGGCATTGTGGAAGG ATCCACAGTCTTCTGGGTGG	69

### Chemotaxis

The migration of IgE-sensitized BMMCs towards Ag as chemoattractant was determined in a 24-well Transwell system with inserts containing polycarbonate filters having 8-μm diameter pores (Corning). TNP-specific IgE-sensitized BMMCs (0.3×10^6^) in 120 µl of chemotaxis medium (RPMI-1640 supplemented with 20 mM HEPES, pH 7.4 and 1% BSA) were added into each Transwell insert and Ag (250 ng/ml TNP-BSA) in 600 µl of chemotaxis medium was added into lower wells of the Transwell system. Cells passing through the polycarbonate filter were counted 8 hours later in 50 µl aliquots with Accuri C6 flow cytometer.

### Statistical analysis

Statistical significance of differences was evaluated by Student's t-test, except for microarray gene-expression profiling in which intergroup differences were evaluated by ANOVA test.

## Results

### Efficient NTAL KD in BMMCs

**Figure 1:**
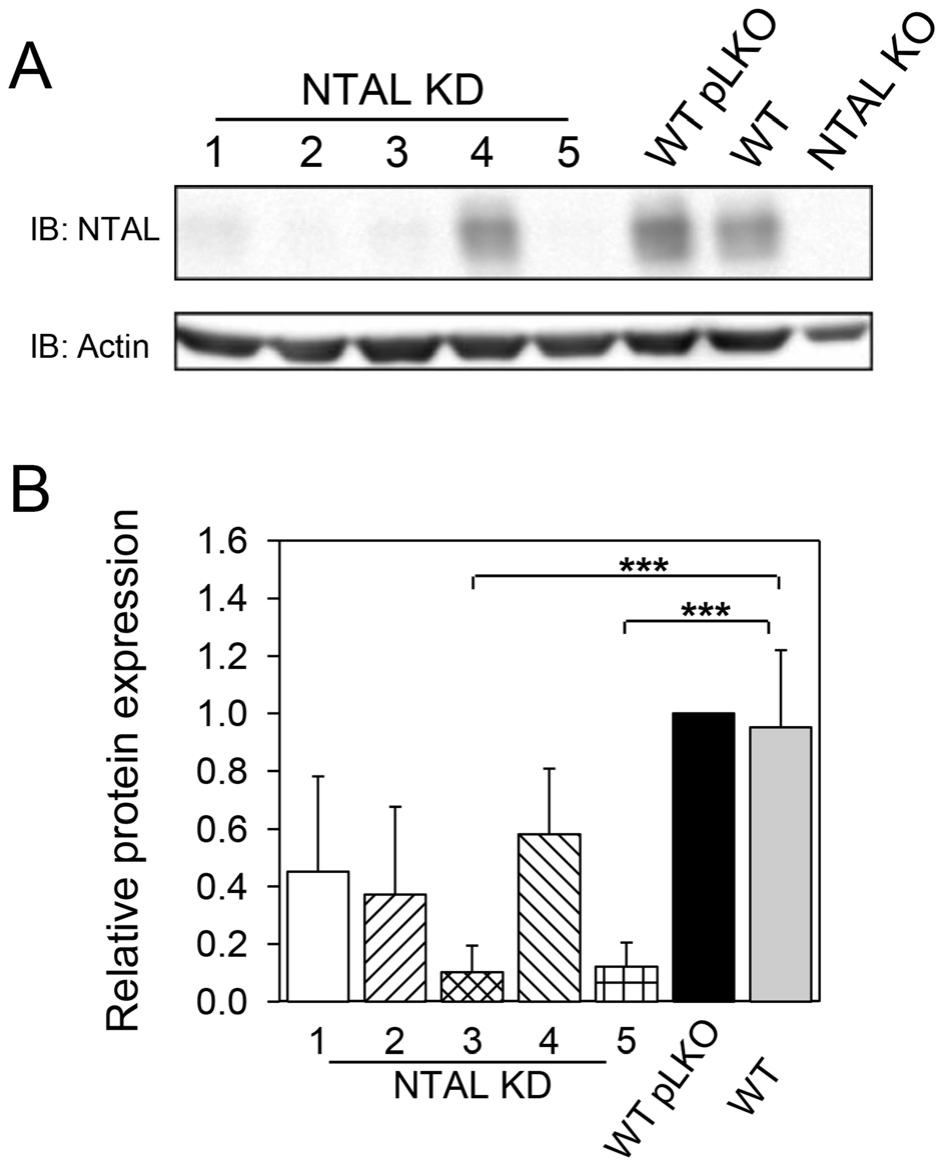
Decreased NTAL expression after shRNA silencing. (A) BMMCs were infected with five lentiviral shRNA constructs (NTAL KD 1–5) or empty pLKO.1 construct (WT pLKO). After selection in puromycin, the amount of NTAL was assessed by immunoblotting. For comparison, NTAL in noninfected WT and NTAL KO cells was also evaluated. Actin was used as a loading control. (B) Densitometry analysis of NTAL immunoblots. The data were normalized to the amount of NTAL in WT pLKO cells and that of actin. Means ± SD were calculated from 3–7 independent experiments. ***p<0.001.

In order to obtain mast cells with stable reduction of NTAL expression by KD approach, five different shRNAs were introduced into *Ntal* ^+/+^ BMMCs by lentiviral-mediated infection, followed by selection in puromycin. To minimize the effect of variables other than the presence of NTAL, target *Ntal* ^+/+^ cells were the same as those which served as WT littermate controls to BMMCs isolated from *Ntal* ^-/-^ mice (KO). Controls for NTAL KDs were the same *Ntal* ^+/+^ BMMCs infected with empty pLKO.1 vector and selected in puromycin. Immunoblotting with NTAL-specific monoclonal antibody confirmed that all five shRNAs inhibited NTAL expression to different degrees (Figure 1A, B). Two of them, namely NTAL KD 3 and NTAL KD 5, showed the highest (>90%), reproducible and highly significant inhibition of NTAL expression and were therefore selected for further experiments. No decrease in NTAL expression was observed in cells infected with empty pLKO.1 vector (WT pLKO; Figure 1A, B). Flow cytometry analysis showed that BMMCs with NTAL KD expressed FcεRI (>95% cells positive) and KIT (>95% positive) at levels comparable to those in WT cells and WT pLKO cells (not shown).

### NTAL KD results in enhanced degranulation and Ca^2+^ response

**Figure 2:**
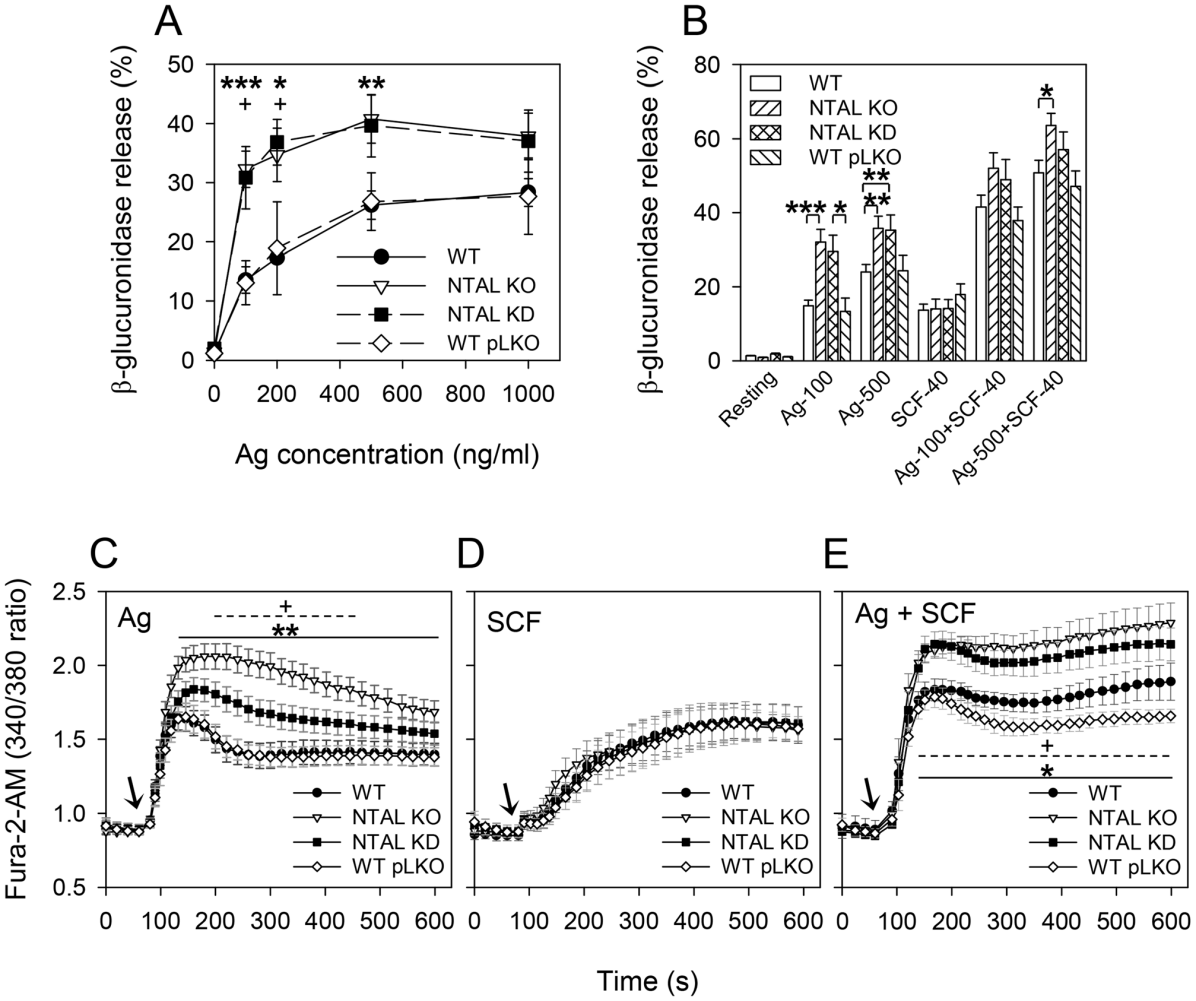
BMMCs with NTAL KD exhibit increased degranulation and calcium response. (A) IgE-sensitized BMMCs (WT, NTAL KO, NTAL KD, and WT pLKO) were stimulated for 30 minutes with various concentrations of Ag (TNP-BSA), and β-glucuronidase released into supernatant was determined as described in Materials and Methods. Data represent means ± SE from 7–17 independent experiments performed in duplicates or triplicates). (B) IgE-sensitized BMMCs were stimulated with Ag [TNP-BSA at a concentration 100 ng/ml (Ag-100) or 500 ng/ml (Ag-500)], SCF (40 ng/ml), or both activators together. Data represent means ± SE from 6–20 independent experiments). (C–E) BMMCs were sensitized with IgE, loaded with Fura-2-AM (1 µg/ml), then stimulated with Ag (100 ng/ml TNP-BSA; C), SCF (40 ng/ml; D) or both activators together (E) and free intracellular Ca^2+^ was monitored by measuring fluorescence emission at 510 nm after excitation at 340 and 380 nm. Arrows indicate addition of Ag and/or SCF. Data are means ± SE from 11 (C), 7 (D) or 6 (E) independent experiments performed in duplicates. All data presented in A–E were obtained with BMMCs isolated from 3–5 mice. *^,+^ *p*<0.05; **p<0.01; ***p<0.001; in A, C and E, significant differences between NTAL KOs and WTs (asterisks) and NTAL KDs and WT pLKOs (crosslets) are shown.

As described in Introduction, there are conflicting reports on the role of NTAL in mast cell signaling in mammalian systems. To address these discrepancies, we compared under well-defined conditions the effect of NTAL KO and KD on mast cell signaling. First we evaluated degranulation. The cells were sensitized with IgE and stimulated with various concentrations of Ag. Degranulation was then determined as the amount of β-glucuronidase released into the supernatant. Data presented in Figure 2A indicate that BMMCs with both NTAL KD and NTAL KO showed enhanced degranulation when compared to the corresponding controls (WT pLKO and WT). The difference between NTAL-deficient cells (KD or KO) and controls (WT pLKO and WT) was more pronounced at suboptimal concentrations of Ag (100 and 200 ng/ml) and was not significant at supraoptimal concentration (1000 ng/ml). Our results show a similar trend for both NTAL KD and KO BMMC. To check for possible off-target effects of lentiviral infection and puromycin selection we also examined antigen-induced degranulation of NTAL KO cells transduced with NTAL shRNA vectors and found no significant difference between infected and noninfected cells (data not shown).

It is known that degranulation is enhanced in cells simultaneously triggered via FcεRI and KIT, a receptor for SCF [21]. We found degranulation not only after exposure of the cells to IgE-antigen complexes, but also after triggering with SCF alone. This is probably due to the fact that mast cells were differentiated from their precursors in the presence of IL-3 and SCF [22]. NTAL KO has no effect on degranulation induced by SCF alone and SCF enhances Ag- induced degranulation in both WT and NTAL KO cells [18]. We therefore investigated degranulation of cells with NTAL KD and the corresponding controls activated by Ag and/or SCF. Compared to separate activation by Ag (100 or 500 ng/ml) or SCF (40 ng/ml), simultaneous activation by Ag and SCF raised degranulation in *Ntal* ^+/+^ controls (WT and WT pLKO; Figure 2B). In cells with NTAL KD, the enhanced degranulation induced by Ag was further increased if the cells were simultaneously activated with SCF (Ag + SCF), even though the difference between NTAL-KD and control cells transduced with empty pLKO was not significant. Similar data were obtained in BMMCs from NTAL KO mice.

Calcium mobilization is another hallmark of mast cell activation. We therefore activated IgE-sensitized BMMCs with Ag in the presence of extracellular Ca^2+^ and evaluated calcium mobilization by means of Ca^2+^-sensitive fluorophore Fura-2-AM. Both *Ntal* ^+/+^ controls (WT and WT pLKO) showed a comparable increase in [Ca^2+^]_i_ peaking at 50–60 s after exposure to Ag (Figure 2C). NTAL KO cells showed the expected [9], [10] long-lasting increase in Ca^2+^ mobilization. Within 120–600 s this response significantly (P<0.01) differed from that seen in WT cells. Significant increase in calcium response was also observed in NTAL KDs between 180–450 s. No significant difference in Ca^2+^ response between NTAL-deficient cells and controls was observed after stimulation with SCF (Figure 2D). Exposure of all cell types to a mixture of Ag and SCF resulted in an accelerated increase in the [Ca^2+^]_i_, and again, BMMCs with NTAL KO and KD cells showed higher Ca^2+^ mobilization than the controls, WT and WT pLKO (Figure 2E). These data indicate that negative regulatory roles of NTAL on FcεRI-mediated degranulation and Ca^2+^ response are due to the absence of NTAL rather than possible compensatory developmental changes induced in NTAL KO.

### NTAL depletion and deletion induce tyrosine phosphorylation of ERK and LAT

**Figure 3:**
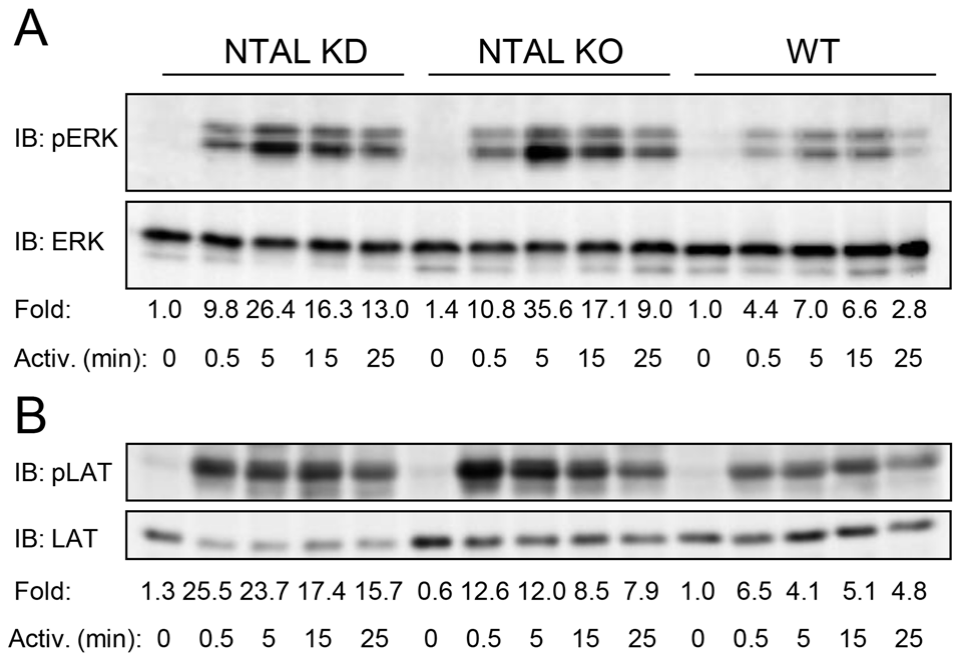
FcεRI-activated BMMCs with NTAL KD show enhanced tyrosine phosphorylation of ERK and LAT. NTAL-deficient cells and WT controls were sensitized with IgE and stimulated with Ag (100 ng/ml TNP-BSA). After the indicated time intervals, the cells were solubilized in lysis buffer and postnuclear supernatants were analyzed by immunoblotting for tyrosine phosphorylated ERK (pERK; A) or LAT (pLAT; B), followed by stripping and immunoblotting for total ERK (A) or LAT (B). Fold inductions of protein tyrosine phosphorylation, normalized to nonactivated WT cells and corrected for the amount of protein in each lysate, are also included. Each immunoblot is a typical result from three independent experiments.

Mast cell activation is initiated by tyrosine phosphorylation of the β and γ subunits of FcεRI, followed by phosphorylation of numerous substrates, including ERK and LAT [23], [24]. It has been suggested that enhanced tyrosine phosphorylation of LAT and some other substrates in *Ntal* ^-/-^ cells could reflect a better accessibility of kinases to LAT in the absence of competition between NTAL and LAT as substrates [9]. This process could be subjected to compensatory developmental alterations. We therefore decided to determine phosphorylation of ERK and LAT in cells with NTAL KD. Immunoblotting experiments showed an increase of tyrosine phosphorylation of ERK (Figure 3A) and LAT (Figure 3B) in NTAL KD cells when compared to WT cells. WT pLKO cells showed similar phosphorylation profile as WT cells (data not shown). Since the same enhanced phosphorylation of LAT and ERK was observed in NTAL KO and NTAL KD cells, developmental compensation mechanisms are unlikely to be responsible for enhanced phosphorylation of the targets.

### Effect of NTAL on cell spreading and chemotaxis

**Figure 4:**
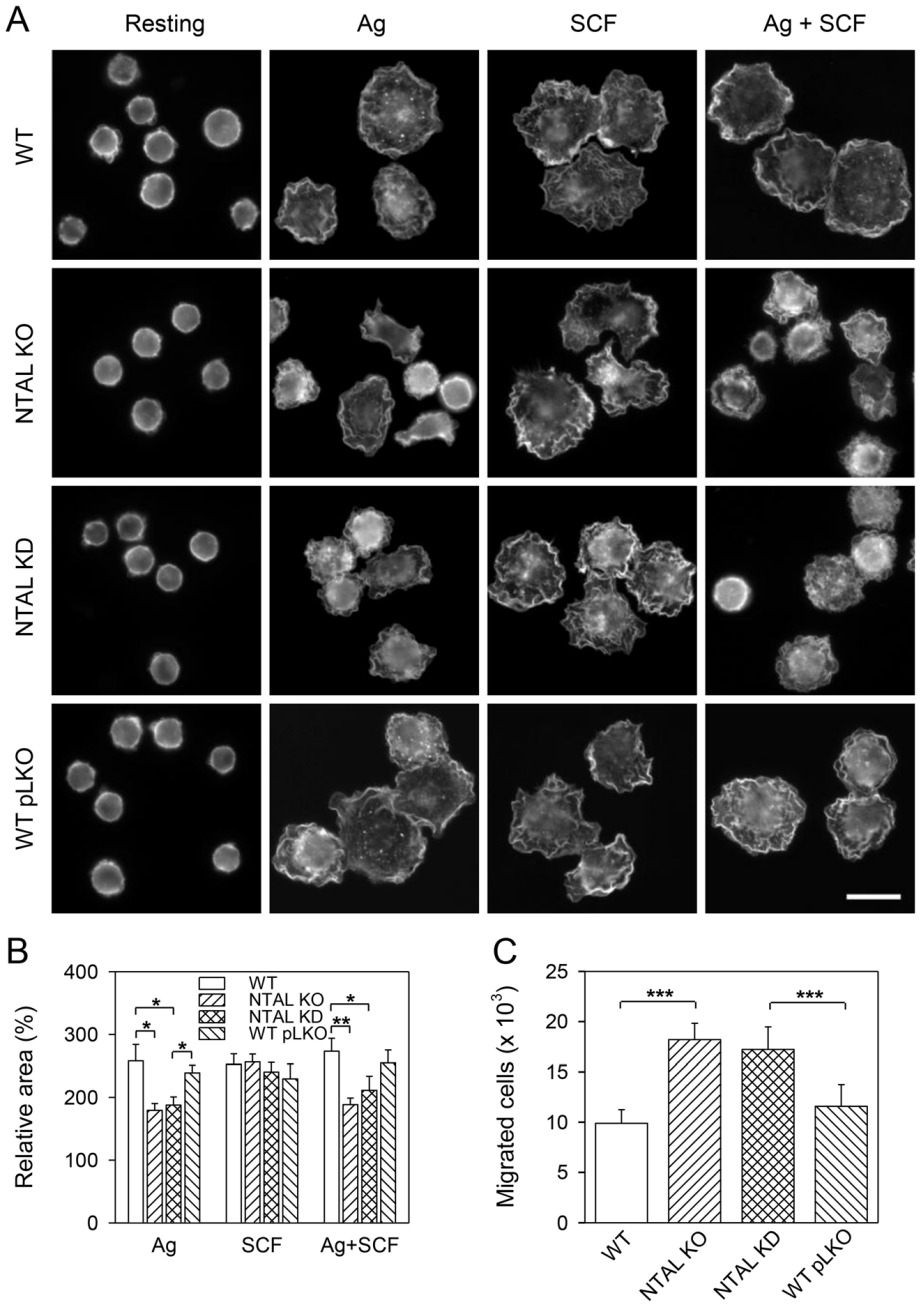
BMMCs with NTAL KD exhibit reduced spreading on fibronectin after stimulation with Ag or Ag + SCF, and enhanced chemotaxis towards Ag. (A) NTAL deficient cells and controls were sensitized with IgE, attached to fibronectin/Cell-Tak–coated glass for 1 hour at 37°C and then challenged or not (Resting) with Ag (250 ng/ml TNP-BSA), SCF (40 ng/ml) or both (Ag + SCF) for 30 minutes. Afterwards the cells were fixed and stained for actin with Alexa Fluor 488 phalloidin. Scale bar 10 µm. All images are depicted in the same scale. (B) Cell areas were determined using ScanR analysis software and data were normalized to nonactivated cells. Means ± SE were calculated from 6 independent experiments with at least 500 cells evaluated in each test. (C) Chemotactic response of IgE-sensitized BMMCs (WT, NTAL KO, NTAL KD and WT pLKO) towards Ag (250 ng/ml TNP-BSA) was evaluated by means of Transwell system with polycarbonate membrane. Numbers of the cells migrating through the membrane after 8 hours were determined by flow cytometry. Means ± SD were calculated from 4 independent experiments performed in duplicates. *p<0.05; **p<0.01; ***p<0.001.

Activation through FcεRI or KIT results in enhanced spreading of BMMCs on fibronectin [25], [26]. Our previous studies with NTAL KO BMMCs showed that full-value spreading on fibronectin was dependent on the presence of NTAL in FcεRI-activated, but not KIT activated, cells [18]. Spreading on fibronectin requires expression of intact integrins and signaling pathways, which could be developmentally regulated. Therefore, we analyzed spreading of BMMC on fibronectin in controls and cells with NTAL KD after exposure to Ag and/or SCF. Data presented in Figure 4A show that in relation to WT and WT pLKO cells, cells with NTAL KD exhibited decreased spreading after activation with Ag. Activation with both Ag and SCF also reduced the spreading of cells with NTAL KD, which showed similar response as cells from NTAL KO mice. No inhibition of spreading was observed in NTAL-deficient cells after activation with SCF. Quantitative analysis of the data obtained is shown in Figure 4B. The area of individual cells was measured and normalized to that of nonactivated cells. Compared to corresponding controls, NTAL KDs and KOs exhibited a significant decrease in surface area after triggering with Ag. Similarly, clear inhibition of cell spreading was observed in both NTAL KOs and KDs activated with Ag + SCF. The difference between NTAL KDs and WT pLKO control, stimulated with Ag + SCF was, however, not significant, mainly because of slight decrease in the spreading of cells with WT pLKO.

We also tested the chemotactic response of NTAL-deficient cells in comparison to WT cells. Data presented in Figure 4C indicate that BMMCs with NTAL KD exhibited significantly enhanced Ag-mediated chemotaxis, similarly as cells with NTAL KO. There was no significant difference between the two cells types.

### NTAL KD increases F-actin depolymerization

**Figure 5:**
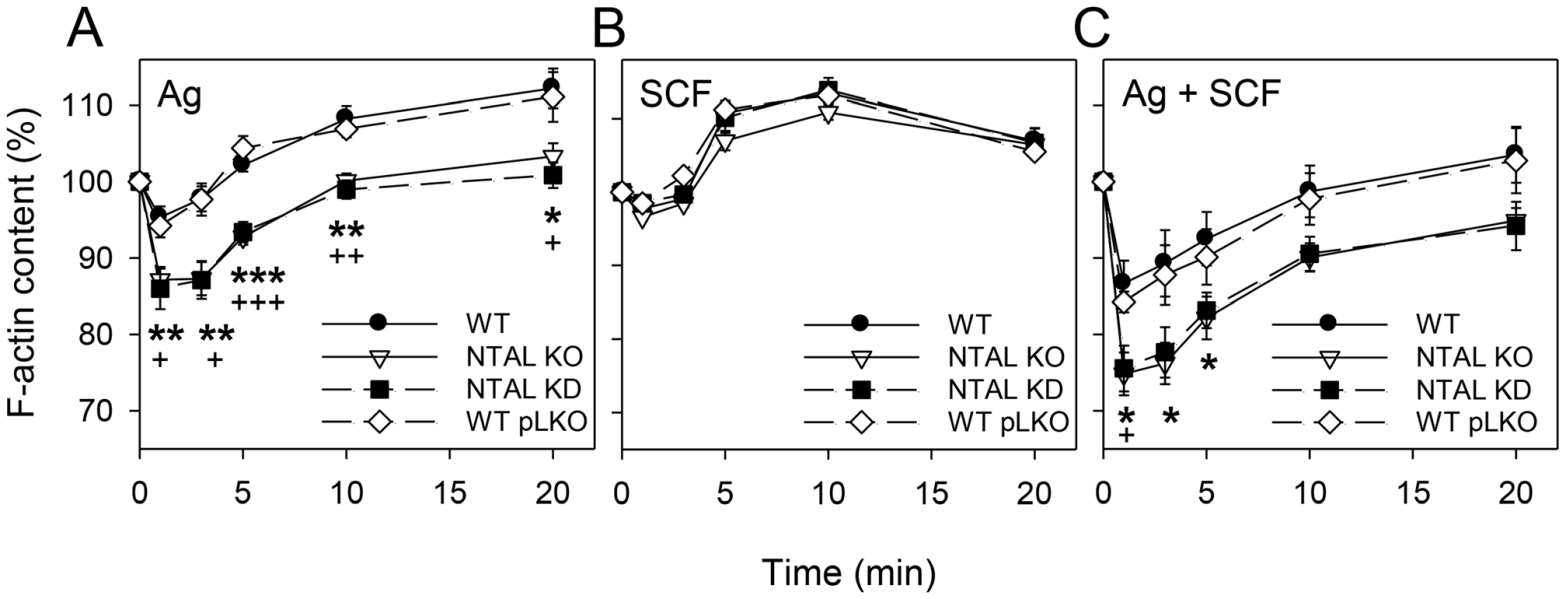
BMMCs with NTAL KD exhibit enhanced actin depolymerization after stimulation with Ag or Ag + SCF. Cells were activated with Ag (250 ng/ml TNP-BSA; A), SCF (40 ng/ml; B) or Ag + SCF (C). At the indicated times, the cells were fixed, stained for F-actin with Alexa Fluor 488-phalloidin and analyzed by flow cytometry. Data were normalized to fluorescence of resting cells (similar in all cell types). Values indicate mean ± SE (n = 6). *^,+^ *p*<0.05; **^,++^ *p*<0.01; ***^,+++^ *p*<0.001; significant differences between NTAL KOs and WTs (asterisks) and NTAL KDs vs WT pLKOs (crosslets) are shown.

FcεRI-induced activation of BMMCs is accompanied by rapid F-actin depolymerization [27]. To determine whether F-actin depolymerization is similarly regulated in NTAL KDs, we activated cells with NTAL KD or NTAL KO and corresponding controls with Ag and/or SCF for the indicated time intervals, and determined the amount of F-actin by flow cytometry. Data presented in Figure 5A show that triggering with Ag stimulated both NTAL KOs and KDs to significantly higher F-actin depolymerization when compared to WT cells. We also observed that SCF activation induced clear increase in F-actin formation, rather than actin depolymerization, and no difference between NTAL KOs and KDs was noticed (Figure 5B). Cells activated by both activators (Ag + SCF) responded by stronger depolymerization than cells activated with Ag alone, and again no difference between cells with NTAL KO and KD was observed (Figure 5C).

### Transcriptome profiles of cells with NTAL KO or KD

To better understand the role of NTAL in mast cell physiology and Ag-induced signaling pathways, we compared gene expression profiles of nonactivated and Ag-activated NTAL-deficient BMMCs with the corresponding controls. Four groups of cells (NTAL KO, NTAL KD, WT, and WT pLKO) were prepared and maintained under comparable culture conditions. Each group consisted of BMMCs isolated from three mice to account for variability of cell donors and procedures of BMMCs isolation. RNA was isolated from IgE-sensitized nonactivated cells or cells activated for 2 hours with Ag. The same RNA was used for microarray analysis and for later confirmation of the gene expression by qPCR. First, we compared expression profiles of nonactivated NTAL KO cells with nonactivated littermate WT controls; from 209 differentially expressed genes (258 probe sets), 70 showed more than 1.8 fold upregulation in NTAL KO cells (Table S1). When focused on biological processes and molecular functions of the genes (Table 2), a large group of the genes involved in metabolic and biosynthetic processes as well as in transcription, translation, and mRNA and rRNA processing was present. The gene list contains about 60 genes with DNA, RNA, or nucleotide binding functions involved in regulation of transcription, replication, and splicing, 21 genes with protein binding function, but, surprisingly, only 6 genes with receptor or signal transducer activity [*Il12rb1* (0.36 fold decrease in KO cells), *Pdcd1lg2*, (3.22 fold increase), *Lgr4* (2.31), *Calca* (2.70), *Lrp8* (0.47) and *Bzrap1* (0.39)]. Other differentially regulated genes are involved in protein phosphorylation and dephosphorylation [*Mlkl* (0.48), *Cdkl2* (2.79), *Ptp4a3* (0.33), *Dusp5* (2.19), *Ppp1r14b* (0.43)] and MAPK kinase kinase cascade [*Map3k7* (0.42) and *Pebp1* (0.49)]. In a group of genes involved in metabolic and biosynthetic processes we found downregulated genes in steroid biosynthetic processes and lipid metabolism.

**Table 2:** Differences in transcriptional regulation between NTAL KO and WT cells.

Biological processes different in NTAL KO vs WT cells			Molecular functions different in NTAL KO vs WT cells		
Biological process	Genes in non-activated cells	Genes in activated cells	Molecular function	Genes in non-activated cells	Genes in activated cells
metabolic processes	24	17	nucleotide binding	38	41
biosythetic processes	15	2	protein binding	21	31
transcription and its regulation	12	15	DNA binding	14	20
mRNA/rRNA processing and translation	11	2	catalytic activity	12	5
transport	11	3	RNA binding	8	1
protein phosphorylation and dephosphorylation	8	9	nucleic acid binding	7	5
cell cycle	7	14	receptor and signal transducer activity	6	3
ATP catabolic process	5	5	binding	5	5
DNA replication	5	6	magnesium ion binding	4	0
apoptosis	4	5	phosphoprotein phosphatase activity	4	2
signal transduction	4	6	chromatin binding	2	0
mitosis	3	9	peptidyl-prolyl cis-trans isomerase activity	2	1
DNA repair	3	7	actin binding	2	3
protein folding	3	1	serine-type endopeptidase inhibitor activity	2	1
microtubule organization and depolymerization	1	4	iron ion binding	2	1
cell proliferation	0	4	calcium ion binding	2	0
ubiquitin-dependent processes	0	4	structural molecule activity	1	2
cytokinesis and cytokine production	0	4	ubiquitin thiolesterase activity	0	2
other process	51	31	other function	44	28
unknown process	42	46	unknown function	33	43
**total number of genes**	**209**	**194**	**total number of genes**	**209**	**194**

When comparing transcriptomes of nonactivated NTAL KD cells with their WT controls, infected with empty pLKO.1 vector, 162 differentially expressed genes (200 probe sets) were identified (Table S2). Some of these genes are involved in protein phosphorylation and dephosphorylation [*Dusp4* (3.55), *Ptpn2* (0.55), *Ptpn3* (1.81) *Dusp5* (2.33), *Epha5* (2.58) and *Mylk3* (0.39)] and signal transduction [*Tmem123* (0.47), *Rasal3* (2.00), *Mmd* (2.70), *Il1r2* (2.90), *Sema4b* (1.81), *Lphn1* (0.42), and *Ogfrl1* (0.52)]. Interestingly, among differentially expressed genes was *Idi*, which was also downregulated in NTAL KO cells.

**Figure 6:**
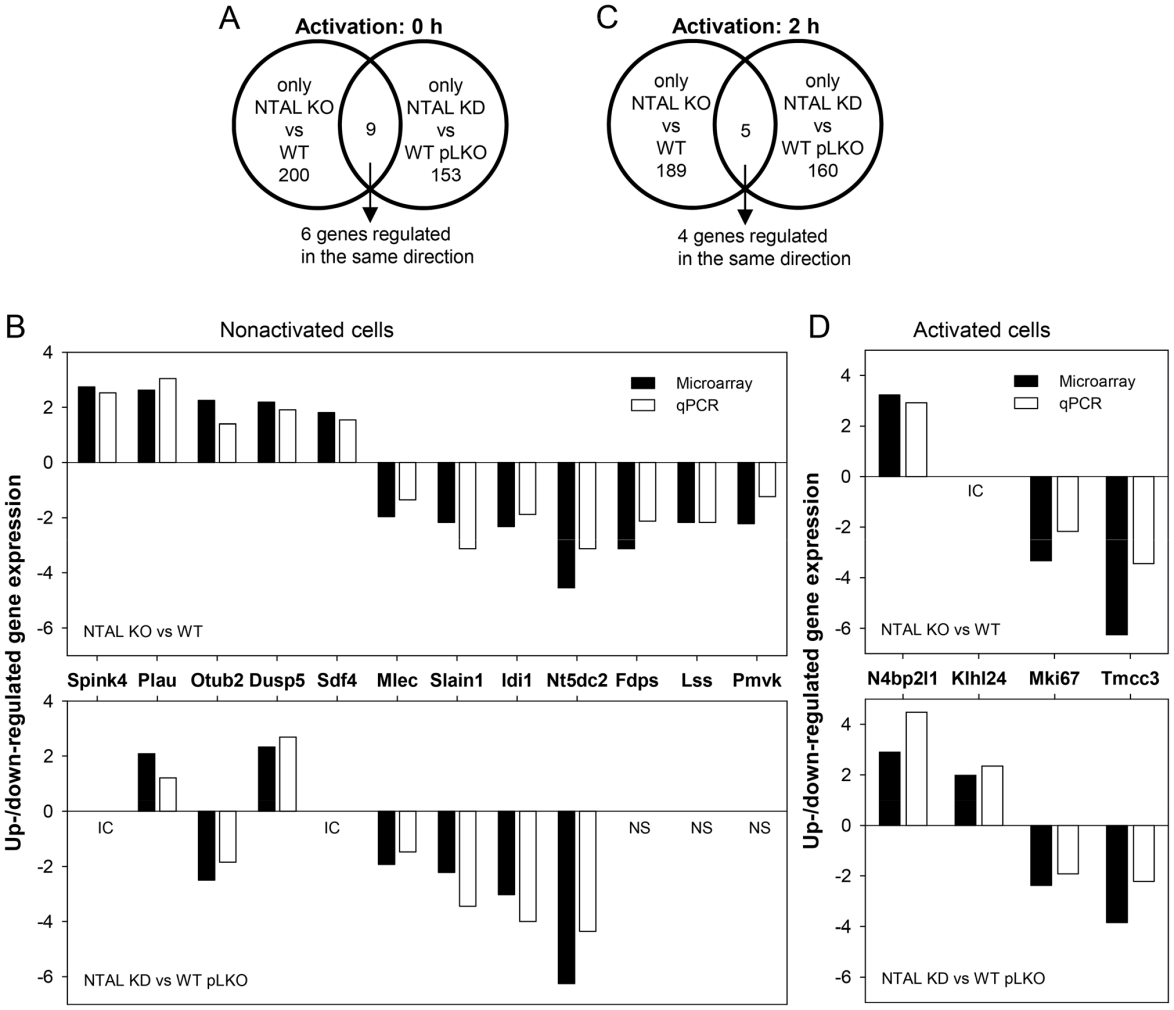
NTAL-dependent up- or down-regulated gene transcripts in resting and Ag-activated BMMCs. (A) Venn diagram illustrating number of genes with expression significantly (>1.8 x) up- or down-regulated in nonactivated cells with NTAL KO vs WT cells and NTAL KD vs WT pLKO cells; only 9 genes showed overlap. (B) Fold up- or down-regulation of overlapping genes in cells with NTAL KO and NTAL KD as determined by microarray data and qPCR analysis. Not shown are genes where qPCR data were inconsistent (IC) with microarray data and data with no significant p-values in microarray data analysis (NS). Four down-regulated genes involved in cholesterol synthesis in NTAL KO cells are also included (*Idi1, Fdps, Lss* and *Pmvk*). (C) Venn diagram as in A but documenting genes in Ag-activated NTAL KO and KD cells; only 5 genes showed overlap. (D) Microarray data and qPCR analysis of the overlapping genes in Ag-activated NTAL KO and KD cells as described in C; only 4 genes showing transcriptional regulation in the same direction are shown.

Numbers of differentially expressed genes in nonactivated NTAL KO and KD cells and their overlaps are schematically depicted in Figure 6A. Expression levels of the overlapping genes were verified by qPCR. The data presented in Figure 6B show that from 9 differentially expressed genes showing overlap between KO and KD, 5 genes were upregulated in NTAL KO cells (*Spink4*, *Plau*, *Otub2*, *Dusp5* and *Sdf4*). Two of them were also upregulated in NTAL KD cells (*Plau* and *Dusp5*); one gene, *Otub2*, was dowregulated in NTAL KD cells, and *Spink4* and *Sdf4* gave in NTAL KD cells results which were not consistent between microarray analysis and qPCR. Downregulated genes involved *Mlec*, *Slain1*, *Idi1* and *Nt5dc2* and were comparably reduced in both NTAL KO and KD cells.

Analysis of gene expression in Ag-activated cells revealed 194 genes (235 probe sets) differentially expressed between NTAL KO cells and WT cells (Table S3). Among them, 83 genes showed more than 1.8 fold upregulation. Most of the genes are involved in transcription, other genes are involved in metabolic processes, production and function of cytokines and in cytoskeleton organization and function. Analysis of Ag-activated NTAL KDs revealed 165 genes (203 probe sets; Table S4).

Numbers of differentially expressed genes in Ag-activated NTAL KO and KD cells and their overlaps are schematically shown in Figure 6C. Expression levels of the overlapping genes were verified by qPCR. Data presented in Figure 6C and D show that from the 5 overlapping genes in activated KD and KO, 4 of them showed transcriptional regulation in the same direction. In NTAL KO cells, *N4bp2I1* gene was upregulated but *KIhI24* gave results which were not consistent between microarray analysis and qPCR. In NTAL KD cells both these genes were upregulated. In both NTAL KO and KD cells *Mki67* and *Tmcc3* were downregulated in both cells types.

**Figure 7:**
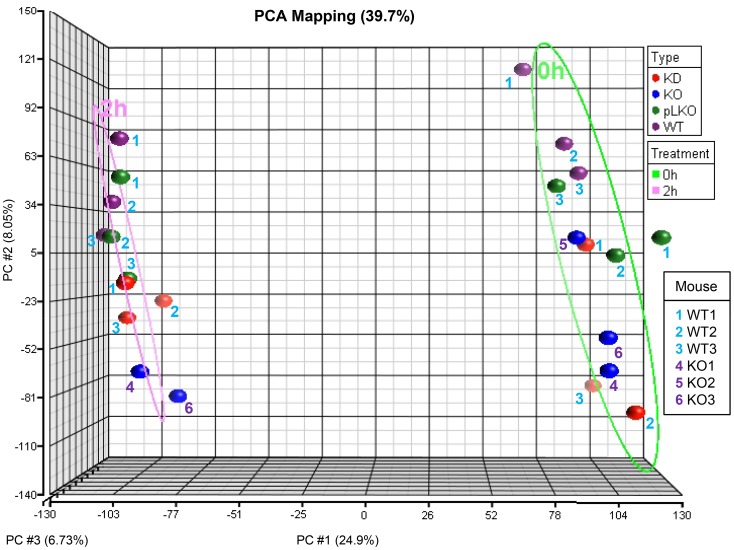
Principal component analysis of the microarrays. Each colored circle represents different cell type: NTAL KD (KD; red), NTAL KO (KO; blue), WT pLKO (pLKO; green), and WT (lilac). Each mouse from which the cells originated is identified by a colored numbers. The WT BMMCs isolated from mice 1–3 (blue numbers) were used not only as controls for NTAL KO cells, but also for obtaining NTAL KD and WT pLKO cells after lentiviral infection with NTAL shRNA or empty pLKO vector, respectively. The BMMC isolated from *Lat* ^-/-^ mice 4–6 (lilac numbers) were used only as cells with NTAL KO. Treatment [Ag-activated cells (2h, pink) and nonactivated cells (0h, green)] is distinguished by ellipsoids. The arrays cluster according to the treatment groups showing separation along PC #1 and according to the type of cells showing separation along PC #2. The percentage values indicate the proportion of total variance described by each PC; PC #1 (X-axis), PC #2 (Y-axis), and PC #3 (Z-axis).

We also looked at changes of gene expression profiles after FcεRI triggering of NTAL KO, NTAL KD, WT and WT pLKO BMMCs. With a more stringent cut-off point of >4 fold up- or down-regulated gene expression and with FDR <0.05, we obtained a list of 308 probe sets representing 244 genes which are shown in Table S5. It is noteworthy that when performing PCA using all probe sets, differences between activated NTAL-deficient cells and the corresponding nonactivated controls were preserved. The highest clustering according to the treatment was found for the first principal component (PC; Figure 7; PC#1) demonstrating that activation of mast cells is a robust process with high impact on transcriptional changes. Smaller clustering was shown according to the type of cells along the second principal component (Figure 7; PC#2).

In this context it was also of interest to determine the contribution of lentiviral infection and selection procedure. We therefore focused on differences in gene expression between WT and WT pLKO cells. In nonactivated cells we found about 100 genes with different expression at the cut-off >1.8 fold change (FDR <0.1), but no difference was found between Ag-activated WT and WT pLKO cells. This was also corroborated by PCA as closer clustering of activated WT and WT pLKO cells (Figure 7).

### NTAL-cholesterol crosstalk in regulation of Ag-mediated chemotaxis

Detailed analysis of the microarray data and gene sorting with the help of Gene Onthology Molecular Function and Biological Process (a module incorporated in the Partek software), suggested that NTAL KO led, among others, to decreased expression of several genes involved in cholesterol synthesis. The genes included isopenthyl-diphosphate delta isomerase1 (*Idi1*), farnesyl diphosphate synthase (*Fdps*), lanosterol synthase (*Lss*) and the phosphomevalonate kinase (*Pmvk*; Table S1 and Figure 6B). Decreased expression of the genes in NTAL KO cells was confirmed by RT-qPCR (Figure 6B). In further experiments we therefore investigated whether NTAL-deficient cells exhibit any change in amount of cellular cholesterol. Using Amplex Red Cholesterol Assay kit we found, however, no significant differences in total amount of cholesterol in both NTAL-deficient cells and corresponding control cells whether the cells were activated or not (data not shown).

**Figure 8:**
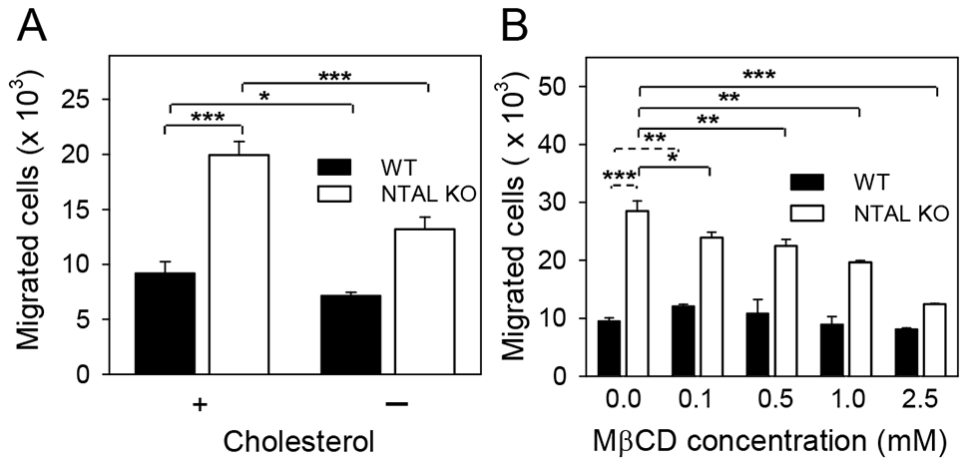
Different sensitivity of Ag-mediated chemotactic response of WT and NTAL KO BMMCs to cholesterol depletion. (A) BMMCs were cultured in media supplemented with IL-3 (without SCF) and 10% FCS with (+) or without (-) cholesterol. After 52 hours the cells were washed, suspended in medium with 10% FCS or cholesterol-depleted FCS and sensitized with TNP-specific IgE (1 µg/ml). After 14 hours, the cells were washed in chemotaxis medium and transferred into Transwell inserts, which were then immersed into the wells of the Transwell system with 600 µl of chemoattractant (250 ng/ml TNP-BSA) in chemotaxis medium. The number of cells passing through the filter was counted 8 hours later with flow cytometer. (B) The cells were cultured in medium supplemented with 10% FCS and IL-3 for 52 hours and then sensitized with TNP-specific IgE (1 µg/ml). After 14 hours, the cells were washed in chemotaxis medium containing various concentrations of MβCD and incubated at 37°C. After 30 min, the cells were washed in chemotaxis medium and transferred into Transwell inserts, which were then immersed into the wells of the Transwell system with 600 µl of chemoattractant (250 ng/ml TNP-BSA) in chemotaxis medium. The number of cells passing through the filter was counted 8 hours later with flow cytometer. Means and SD were calculated from data obtained in 3–4 independent experiments performed in duplicates. *p<0.05; **p<0.01; ***p<0.001.

Experiments with macrophages showed that local redistribution of cholesterol from inner to outer leaflet of the plasma membrane is of key significance for chemotaxis [28]. We therefore compared chemotaxis of NTAL-deficient and control cells cultured for 66 h in media supplemented with FCS or cholesterol-depleted FCS. This latter approach has been previously shown to decrease cholesterol level in BMMCs by ∼25% ([29] and our unpublished data). We found (Figure 8A) that if WT cells grew in media containing cholesterol-deprived FCS, they exhibited lower chemotaxis towards antigen than cells cultured in cholesterol-containing medium (decrease to 78.8%±10.5%, mean ± SD; n = 8). When NTAL KO cells were used the inhibitory effect of cholesterol deprivation was more pronounced (decrease to 66.8%±7.1%, mean ± SD; n = 8). The observed difference in chemotaxis decrease between WT cell and NTAL KO cells was significant (P = 0.004). These data indicate that chemotaxis of NTAL-deficient cells is more sensitive to decreased cholesterol levels than chemotaxis of WT cells.

In parallel experiments we compared chemotaxis of NTAL-deficient and control cells after treatment with various concentrations of methyl-β-cyclodextrin (MβCD), a compound which has been previously shown to reduce cellular cholesterol in mast cells [14], [30]. In accord with previous findings (Figure 4C), Ag-driven chemotactic response was higher in NTAL-deficient cells than that of WT cells (Figure 8B). When NTAL KO BMMCs were exposed to increasing concentrations of MβCD (0.1–2.5 mM), significant decrease in chemotactic response was observed at all concentrations of MβCD tested (Figure 8B). In contrast, WT cells showed no decrease in chemotaxis after exposure to 0.5–2.5 mM MβCD. When exposed to 0.1 mM MβCD even a small but significant increase in chemotactic response to Ag was observed in WT BMMCs. These data suggest that low concentrations of MβCD change distribution of the plasma membrane cholesterol in NTAL KO cells in such a way that their chemotactic response is reduced.

## Discussion

This study was initiated because of long-standing discrepancies in published data indicating that NTAL in mouse mast cells is a negative regulator of FcεRI signaling [9], [10], whereas in human or rat mast cells is a positive regulator [12], [13]. However, it was not clear whether these discrepancies reflect different methods/strategies used for NTAL down-regulation (NTAL KO in mice, whereas NTAL KD in human and rat mast cells) and developmental alterations in KO mice as described in other systems where absence of a given gene is compensated for by enhanced transcriptional activity of other genes [31]–[34]. In attempt to understand the contribution of the compensatory mechanisms, we investigated for the first time the properties of mouse BMMCs with NTAL KD and compared them with BMMCs from mice with NTAL KO and well-matched controls. Several lines of evidence obtained in this study, indicate expressive similarities between the properties of BMMCs with NTAL KD or KO, and support the concept that NTAL is mostly a negative regulator of FcεRI signaling, independently of possible compensatory developmental alterations.

First, BMMCs with both NTAL KO and NTAL KD showed comparable increase in degranulation induced by FcεRI triggering. Compared to WT cells, NTAL KDs showed the highest increase in degranulation at suboptimal concentrations of Ag, similarly to NTAL KOs. At optimal and supraoptimal Ag concentrations the differences were less pronounced. Interestingly, activation through KIT was not potentiated by the absence of NTAL, even though NTAL is tyrosine phosphorylated in KIT-activated mast cells [12], [35] and activation through KIT enhances degranulation of FcεRI-activated WT cells, and even more so of cells with NTAL KO or KD.

Second, Ag-activated BMMCs with NTAL KD exhibited higher Ca^2+^ response when compared to WT pLKO cells, but lower when compared to NTAL KO cells. Similarly to degranulation, down-regulation of NTAL had no effect on Ca^2+^ response after KIT triggering, even though KIT activation enhanced Ca^2+^ response in Ag-activated WT cells, and even more so in NTAL-deficient cells.

Third, when compared to WT cells, Ag activation of cells with NTAL KD resulted in enhanced tyrosine phosphorylation of ERK and LAT. Similar enhancement was also observed in Ag-activated NTAL KOs ([9], [10] and this study). These data support the hypothesis that competition between NTAL and LAT as kinase substrates could attenuate the response in WT cells through decreased tyrosine phosphorylation of LAT, followed by decreased binding and activation of phospholipase Cγ1 and subsequent events [6], [9], [36].

Fourth, BMMCs with both NTAL KD and NTAL KO exhibited enhanced F-actin depolymerization after stimulation with Ag alone and even more after simultaneous triggering with both Ag + SCF. F-actin depolymerization precedes degranulation [27], [37] and the observed decrease in amount of F-actin could account for the observed higher degranulation in NTAL-deficient cells than in WT cells after simultaneous activation with Ag + SCF.

Fifth, cells activated through FcεRI or KIT exhibited enhanced spreading on fibronectin. In cells with NTAL KD spreading was significantly decreased after activation with antigen, but was unaffected after SCF triggering. The observed data suggest that positive regulatory role of NTAL on Ag-mediated spreading ([18] and this study) is not the result of developmental compensatory events. Rather, spreading could be related to transient actin depolymerization which was observed in Ag-activated WT cells and even more in NTAL-deficient cells, but not in SCF-activated cells, WT or NTAL-deficient.

Sixth, BMMCs with NTAL KD exhibited migration towards Ag comparable with that seen in NTAL KO cells, and significantly higher than in WT cells. We recently showed that the level of active RhoA in resting NTAL KO BMMCs is at least twice as high as in WT cells [18]. Although active RhoA transiently decreased after FcεRI triggering, more in NTAL KO cells than in WT cells, it is likely that differences in regulation of RhoA activity in NTAL-deficient cells and WT cells are responsible for the enhanced NTAL-regulated chemotaxis. It should be stressed that previous reports have shown that RhoA regulates chemotaxis in other cell types, such as neutrophils [38]–[40], macrophages [41], dendritic cells [42] and lymphocytes [43].

The data presented in this study, together with those obtained in mice experiencing systemic anaphylaxis [9] indicate that in mouse mast cells NTAL is a negative regulator of FcεRI signaling. In contrast to mouse cells, NTAL in human mast cells and rat basophilic leukemia (RBL)-2H3 cells was described as a positive regulator of mast cells signaling [12], [13], [35]. The observed differences could have several causes. Thus, NTAL could play different roles in mast cells of different origin. It has been shown that human mast cells differ from mouse mast cells in cytokine production, immunoglobulin receptor expression, and the ability of different stimuli to cause degranulation and release of mediators [44]. Furthermore, when total tyrosine phosphorylated proteins were compared between RBL-2H3 cells and freshly isolated peritoneal and pleural rat mast cells, dramatic differences were observed [45]. These differences could reflect tumor origin of RBL-2H3 cells and could be responsible for the observed properties of NTAL. Importantly, mouse and human mast cells were obtained after differentiation under different cell culture conditions, which could modify their responsiveness. Mouse BMMCs were obtained by culturing bone marrow precursors in the presence of IL-3 and SCF (this study; [9]) or IL-3 alone [10], whereas human mast cells were derived from CD34+ pluripotent peripheral blood progenitors cultured in the presence of human SCF, IL-6 and IL-3 [12], [35]. Previous study showed that differentiation of mast cells from their precursors in the presence of various cytokines could result in different responsiveness of the cells to various activators [22]. Finally, one cannot exclude the possibility that silencing vectors used for NTAL KD in human and/or RBL-2H3 mast cells exhibited off-target effects, which modified responsiveness of the cells to FcεRI triggering.

To clarify the role of NTAL in FcεRI signaling and to find out whether absence or decreased expression of NTAL has any effect on transcriptional regulation of genes, we compared under thoroughly controlled conditions RNA expression profiles of resting and Ag-activated BMMCs with NTAL KO or KD and the corresponding controls. We found that number of genes were up- or down-regulated, in BMMCs with NTAL KO or KD when compared to WT cells; most of the genes were not related to known immunoreceptor signaling pathways. The exact mechanisms and pathways through which NTAL causes changes in transcription of these genes remains to be determined. As expected, FcεRI activation induced robust changes in gene expression in all four types of mast cells studied (NTAL KO, NTAL KD, WT and WT pLKO). At the given cut-off level (>1.8-fold difference from proper controls), 209 genes showed different expression in nonactivated NTAL KO cells. It is remarkable that no differences in gene expression were noticed between Ag-activated WT and WT pLKO when similar criteria for analysis of differential gene expression were used. This confirms that infection and puromycin selection had no significant effect on the data obtained from lentivirally infected and activated cells. This is in marked contrast with comparison of RNA from activated cells with NTAL KO vs WT and NTAL KD vs WT pLKO, where 194 and 165 genes, respectively, were found differentially expressed.

When comparing expression levels in various cell types we noticed that the degree of overlap between nonactivated and activated NTAL KO and KD cells was rather modest. This could be due to methodological differences in production of NTAL-deficient cells. However, it should be kept in mind that although lentiviral infection itself and puromycin selection caused differential expression of some genes, as can be deduced from the observed differences in gene expression between nonactivated WT and WT pLKO cells, this difference disappeared in activated cells. Thus, lentiviral infection and puromycin selection did not contribute to the differences observed, at least in activated cells.

**Figure 9:**
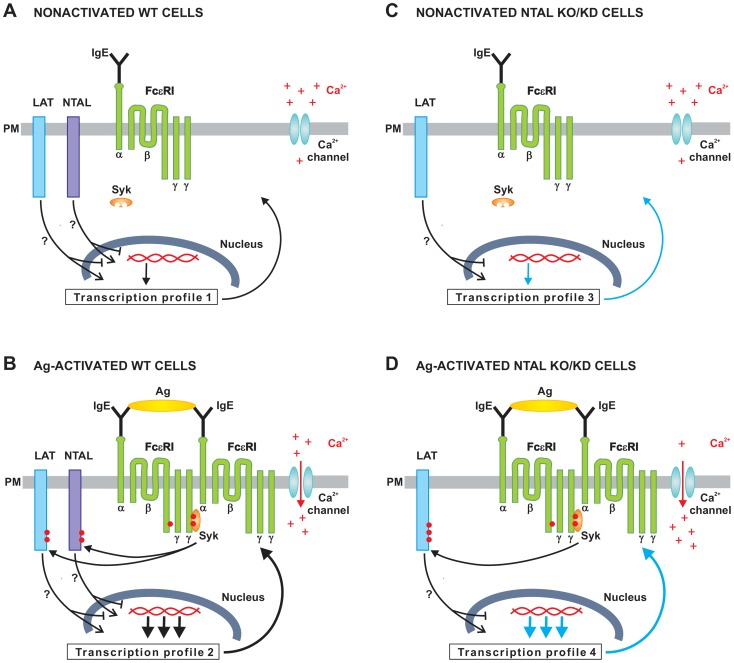
A hypothetical model on the role of NTAL in mast cell activation and transcriptional regulation. (A) In nonactivated WT cells both adaptor proteins, LAT and NTAL, and FcεRI β and γ subunits only exhibit weak phosphorylation, low [Ca^2+^]_i_ and transcription corresponding to nonactivated cells (Transcription profile 1). (B) After Ag-mediated aggregation of the FcεRI-IgE complex, β and γ subunits of the FcεRI are tyrosine phosphorylated by LYN and SYK. SYK then phosphorylates NTAL and LAT and this leads to enhanced Ca^2+^ uptake and further propagation of the signal, including dramatic changes in transcriptional regulation (Transcription profile 2). (C) In nonactivated NTAL-deficient cells, LAT and FcεRI subunits are only weakly tyrosine phosphorylated and the cells exhibit slightly different transcriptional regulation when compared to WT cells (Transcription profile 3). (D) After FcεRI triggering of NTAL-deficient cells, β and γ subunits of the FcεRI are tyrosine phosphorylated as in B, but because of the absence of NTAL, LAT is more phosphorylated by SYK. This leads to enhanced mobilization of Ca^2+^ and other signaling events and transcriptional regulation which differs from the one in activated WT cells (Transcription profile 4).

A hypothetical simplified model on the role of NTAL in mast cell activation and transcriptional regulation in WT and NTAL-deficient cells is shown in Figure 9. In nonactivated WT cells both adaptor proteins, NTAL and LAT, as well as FcεRI β and γ subunits are only weakly tyrosine phosphorylated, because of the equilibrium between kinases and phosphatases and/or decreased access of the kinase to their substrates [46]. Quiescent cells also exhibit low [Ca^2+^]_i_ and standard gene expression (Transcription profile 1; Figure 9A). After Ag-mediated activation there is enhanced tyrosine phosphorylation of FcεRI β and γ subunits by LYN and SYK kinase. Activated SYK phosphorylates NTAL and LAT and this leads to further propagation of the activation signal, increased [Ca^2+^]_i_ and dramatic changes in gene expression by so far not fully understood mechanism (Transcription profile 2; Figure 9B). In cells with decreased expression of NTAL due to NTAL KO or NTAL KD, gene expression is changed when compared to WT cells (Transcription profile 3; Figure 9C). After activation of NTAL-deficient cells, LAT is phosphorylated by SYK. However, because of NTAL absence, LAT is more phosphorylated than in WT cells. This leads to increased [Ca^2+^]_i_ and transcriptional regulation which is different from WT cells (Transcription profile 4; Figure 9D). These processes contribute to enhanced response to Ag in NTAL-deficient cells, including enhanced degranulation, calcium response, chemotaxis and depolymerization of F-actin.

Unexpected findings in this study were NTAL-dependent changes in the expression of a number of genes related to metabolism and biosynthetic processes. A subgroup of these genes was involved in lipid metabolism, including synthesis of cholesterol. Although decreased transcription of several genes involved in cholesterol synthesis was confirmed by RT-qPCR, no significant difference in total amount of cellular cholesterol was detected between WT and NTAL-deficient cells. Yet, surprisingly, we found that pretreatment of BMMCs with MβCD had different effect on NTAL KO and WT cells. In NTAL KO cells MβCD significantly inhibited chemotaxis at all concentrations of MβCD tested (0.1–2.5 mM), whereas in WT cells MβCD either slightly, but reproducibly increased chemotaxis at a low concentration (0.1 mM) or had no significant effect at higher concentrations (0.5–2.5 mM). MβCD is known to remove cholesterol from the cells [47], [48] and therefore one can hypothesize that enhanced chemotaxis in NTAL-deficient cells is regulated in part by plasma membrane cholesterol distribution. Molecular mechanism of the cholesterol-dependent regulations of chemotaxis is poorly understood, but could be related to differences in synthesis and/or distribution of cholesterol into plasma membrane sheets. One such possible mechanism has been recently described in macrophages with defect in ATP-binding cassette transporters ABCA1 and ABCG1, which are involved in the movement of cholesterol from the inner to the outer leaflet of the plasma membrane and play role in chemotaxis towards C5a chemoattractant [28]. Regulation of chemotactic response by cholesterol has been described in other cell types including T cells [49], monocytes [50] and neutrophils [51]. Molecular mechanisms of the cross-talk between NTAL and cholesterol remains to be determined.

In summary, the results based on functional studies of BMMCs with NTAL KD and the corresponding controls indicate that NTAL is a negative regulator of FcεRI-mediated signaling pathways. Because similar findings were observed in BMMCs with NTAL KD or KO, no significant role of compensatory developmental alterations appear to account for FcεRI signaling in BMMCs from *Ntal* ^-/-^ mice. Expression profiles of nonactivated or FcεRI activated BMMCs with NTAL KO, NTAL KD, and the corresponding controls identified several genes which were up- or down-regulated in NTAL-deficient cells. The data indicate that some of these genes could be involved in regulation of cholesterol-dependent events in Ag-mediated chemotaxis.

## Supporting Information

Table S1 Differentially expressed gene transcripts in nonactivated NTAL KO cells when compared with nonactivated WT cells. The table represents a list of probe sets for the corresponding genes that were up- or down-regulated in nonactivated (0 h) NTAL KO cells (KO) when compared to corresponding nonactivated WT cells (WT) and passed the filter of FDR <0.1 and 1.8 fold change (ratio). Probe sets are sorted in ratio descending order. Those probe sets that also show significant up- or down-regulation in noactivated NTAL-KD cells are in bold. For comparison purposes (in grey) are shown p-values and ratios of the selected probe sets from comparison of nonactivated NTAL KD cells (KD) vs nonactivated WT pLKO cells (pLKO), activated (2 h) NTAL KO cells vs activated WT cells, and activated NTAL KD cells vs activated WT pLKO cells.
(XLSX)

Table S2 Differentially expressed gene transcripts in nonactivated NTAL KD cells when compared with nonactivated WT pLKO cells. The table represents a list of probe sets for the corresponding genes that were up- or down-regulated in nonactivated NTAL KD cells when compared to the corresponding nonactivated WT pLKO cells and passed the filter of FDR <0.1 and 1.8 fold change (ratio). Probe sets are sorted in ratio descending order. Those probe sets that also show significant up- or down-regulation in NTAL-KO cells are in bold. For comparison purposes (in grey) are shown p-values and ratios of the selected probe sets from comparison of nonactivated NTAL KO cells vs nonactivated WT cells, activated NTAL KO cells vs activated WT cells, and activated NTAL KD cells vs activated WT pLKO cells.
(XLSX)

Table S3 Differentially expressed gene transcripts in Ag-activated NTAL KO cell when compared with Ag-activated WT cells. The table represents a list of probe sets for the corresponding genes that were up- or down-regulated in Ag-activated NTAL KO cells when compared to the corresponding activated WT cells and passed the filter of FDR <0.1 and 1.8 fold change (ratio). Probe sets are sorted in ratio descending order. Those probe sets that also show significant up- or down-regulation in NTAL KD cells are in bold. For comparison purposes (in grey) are shown p-values and ratios of the selected probe sets from comparison of activated NTAL KD cells vs activated WT pLKO cells, nonactivated NTAL KO cells vs nonactivated WT cells, and nonactivated NTAL KD cells vs nonactivated WT pLKO cells.
(XLSX)

Table S4 Differentially expressed gene transcripts in Ag-activated NTAL KD cells when compared with Ag-activated WT pLKO cells. The table represents a list of probe sets for the corresponding genes that were up- or down-regulated in Ag-activated NTAL KD cells when compared to the corresponding WT pLKO cells and passed the filter of FDR <0.1 and 1.8 fold change (ratio). Probe sets are sorted in ratio descending order. Those probe sets that also show significant up- or down-regulation in NTAL-KO cells are in bold. For comparison purposes (in grey) are shown p-values and ratios of the selected probe sets from comparison of activated NTAL KO cells vs activated WT cells, nonactivated NTAL KO cells vs nonactivated WT cells, and nonactivated NTAL KD cells vs nonactivated WT pLKO cells.
(XLSX)

Table S5 Differentially expressed gene transcripts in all four groups of cells after Ag activation when compared to their noinactivated forms. The table represents a list of probe sets for the corresponding genes that were up- or down-regulated among all four groups of cells when the same Ag-activated (2 h) and nonactivated (0 h) cells were compared. Table shows probe sets that passed the filter of FDR <0.05 and 4 fold change (ratio). Probe sets are sorted in ratio descending order. Correspondig unadjusted p-values and ratios of these probe sets from comparison of activated WT cells vs nonactivated WT cell, activated NTAL KO cells vs nonactivated NTAL KO cells, activated NTAL KD cells vs nonactivated NTAL KD, and activated WT pLKO cells vs nonactivated WT pLKO cell are shown.
(XLSX)
